# A longitudinal follow-up of posttraumatic stress: from 9 months to 20 years after a major road traffic accident

**DOI:** 10.1186/1753-2000-5-8

**Published:** 2011-03-11

**Authors:** Filip K Arnberg, Per-Anders Rydelius, Tom Lundin

**Affiliations:** 1 National Centre for Disaster Psychiatry, Department of Neuroscience, Uppsala University, Uppsala, Sweden; 2 Department of Women's and Children's Health, Karolinska Institutet, Stockholm, Sweden

## Abstract

**Background:**

Although road traffic accidents (RTA) are a major cause of injury and a cause of posttraumatic stress (PTS) in the aftermath, little is known about the long-term psychological effects of RTA.

**Methods:**

This prospective longitudinal study assessed long-term PTS, grief, and general mental health after a bus carrying 23 sixth-grade schoolchildren crashed on a school outing and 12 children died. Directly affected (i.e., children in the crash) and indirectly affected children (i.e., all pupils in the sixth grade who were not in the crash) were surveyed at 9 months (*N *= 102), 4 years (*N *= 51), and 20 years (*N *= 40) after the event. Psychological distress was assessed by single items, including sadness, avoidance, intrusions, and guilt. After 20 years, PTS was assessed by the Impact of Event Scale-Revised.

**Results:**

Stress reactions were prevalent 9 months after the event, with sadness (69%) and avoidance (59%) being highly represented in both directly and indirectly affected groups, whereas, nightmares (60%) and feelings of guilt (50%) were only frequent in those directly affected. The frequency of sadness and avoidance decreased after 4 years in the indirectly exposed (*p*s < .05). After 20 years, the directly affected had a higher prevalence of PTS (*p *= .003), but not decreased general mental health (*p *= .14), than those indirectly affected.

**Conclusions:**

The limitations preclude assertive conclusions. Nonetheless, the findings corroborate previous studies reporting traumatic events are associated with long-term PTS, but not with decreased general mental health.

## Background

Road traffic accidents (RTA) are a major cause of injuries and deaths. In traffic, children are a particularly vulnerable group. In Sweden, 30 100 children (i.e., 1 561 per 100 000) aged 0-17 years attended an Accident and Emergency Department during 2008 due to RTA [1]. In addition to physical injuries, children involved in RTA may experience posttraumatic stress [2,3]. Posttraumatic stress includes symptoms of re-experiencing, such as flashbacks and nightmares; avoidance of reminders of the event and emotional numbing; and increased arousal manifested in hyper vigilance, jitteriness and concentration difficulties. If posttraumatic stress symptoms (PTSS) persist for over one month and cause significant distress or impairment in functioning, the diagnosis posttraumatic stress disorder (PTSD) is warranted [4]. PTSD is only one consequence of RTAs, other psychological effects include travel anxiety and phobic anxiety disorder [5,6], and depression and generalised anxiety disorder [2,7]. Posttraumatic stress disorders were first applied to children in DSM-IIIR in 1987 [8]. However, little is published on the long-term psychological consequences in children after RTA: A 2009 review of PTSD and PTSS in children after RTA found no studies have assessed posttraumatic stress beyond 18 months [9].

Cognitive and behavioural theories on the development and course of posttraumatic stress [10,11] propose painful intrusions and hyperarousal can establish cognitive processes and behavioural patterns with the purpose of avoiding trauma-related stimuli. The avoidance subsequently maintains the PTSS by precluding the mental processing of emotions and cognitions needed for integrating the experience into a person's own pre-existing system of beliefs and behaviour [10,11]. Consequently, if a configuration regarding responses to trauma-related stimuli is set, any greater accommodation of these responses is unlikely without the processing of the core emotions and cognitions.

In the first weeks after a RTA, PTSS in children and youth can be impairing [12]. In a review of PTSD and PTSS in children after RTA, the prevalence of PTSD is estimated at 30% after 1-2 months [9], and after 3-6 months, the estimated prevalence of PTSD is 13% [9]. Similarly, within 9 months, 17% of children hospitalised for injuries sustained in a traffic crash qualify for PTSD or subsyndromal PTSD (i.e., fewer moderate or severe symptoms and impairment from symptoms) [12].

Although there is generally a decline in PTSS during the first months after the traumatic event, there are prolonged symptoms among children who do not experience quick relief. An 18-month follow-up of young RTA victims [13] revealed that although the levels of posttraumatic stress decreased from 2-16 days to 12-15 weeks, no change in PTSS was detected from the second assessment to 18 months, when one-third of the victims still displayed moderate or severe PTSD symptoms [13]. A recent study [14] used trajectory-modelling to identify patterns of PTSS in children up to 2 years after an accidental injury, and three distinct trajectory groups were identified: children who were resilient (57%), i.e., who experienced no or few symptoms both immediately and at follow-up; children who recovered quickly (33%); and children with chronic symptoms (10%), i.e., who had high levels of PTSS both immediately and at follow-up. Overall, these empirical findings parallel cognitive and behavioural theories on posttraumatic stress [10,11]. However, the progression from childhood through adolescence and into adulthood raises questions as to whether psychological development and maturation decreases or increases the risk of long-lasting PTSS in individuals who have experienced a single traumatic event in childhood [15].

Literature on long-term PTSS after other single traumatic events is scarce. Between 5 and 8 years after the sinking of a cruise ship, a follow-up of survivors (age 11-17 years at disaster) found 52% developed PTSD after the disaster [15], 90% of whom developed PTSD during the first 6 months: the duration of PTSD was > 5 years in 26% of the cases and at follow-up, the point prevalence of PTSD was 34%. These findings suggest PTSS symptoms can persist through adolescence and at least until early adulthood [15]. Morgan et al. [16] found 29% of schoolchildren suffered from PTSD 33 years after a coal slag heap collapsed on to a primary school. However, most long-term follow-up studies are retrospective in design, and as people tend to underestimate past psychiatric problems [17], reliance on retrospective accounts can underestimate the number of participants recovering from PTSS.

Indirectly affected children may also experience PTSS. In a study of seventh grade children after a bus crash on a school outing [18], 39% of children not involved in the crash reported moderate or severe acute stress reactions within the first week; however, after 9 months, only 6% of the same children reported moderate or severe PTSS. After 7 years, Tyano et al. [19] followed up of the directly and indirectly affected children then aged 20 years old: the directly affected children experienced more PTSS than indirectly affected children and controls, and exhibited more mental health help-seeking behaviour. However, the directly exposed children did not differ from the indirectly exposed or controls in terms of general distress. The finding directly exposed differ from the indirectly exposed regarding PTSS, but not general distress, was also supported in the 33-year follow-up of survivors (aged 4-11 years) by Morgan et al. [16]. In summary, information is scarce on long-term psychological consequences of major traffic accidents, as well as other single traumatic incidents, in particular for children.

The aim of this study was to describe the psychological effects of a bus crash on all children of the same age in the affected school. Specifically, the objectives were to compare the frequency of psychological stress reactions over time; to assess differences in psychological stress reactions after 9 months between directly and indirectly affected children (i.e., those who were on the bus and those who were not) 20 years after the bus crash; and to compare posttraumatic stress reactions, complicated grief, and decreased general mental health between those directly and indirectly affected.

## Methods

### The Event

In 1988, a tour bus on a school outing had a brake failure inside a tunnel. Onboard were 23 twelve-year old children, 9 parents, and the teacher with her spouse. The driver attempted to decelerate by forcing the bus against the tunnel wall; at the tunnel opening, the bus crashed into a concrete beam. Twelve children and three parents died in the accident: the driver died 12 days later. The majority of survivors had multiple injuries to the head, chest, abdomen, and limbs: no permanent neurological damage was reported. The injured children regained physical mobility within months and resumed regular school attendance from 1 week to 4 months after the accident. Eleven children survived and there were two children in the affected class who had not participated in the school outing. The event received extensive nationwide media coverage and acute support interventions were deployed [20]. Two of the authors of this study (PAR and TL) took active part in the intervention program that followed. The families involved in the bus crash participated in a crisis intervention program during the first 9 days after the accident, and the passengers received psychological treatment during the first 6 months, on average, after the event [20]. The psychological adjustment of the affected adults and families has been previously described [21].

### Procedure

The present study combined a long-term follow-up and an initial follow-up undertaken by the authors PAR and TL. The results of the initial study have not been published. In their initial study, all sixth-grade pupils (*N *= 107) in the affected school on that day were distributed a questionnaire by their teachers nine months after the bus crash. After 4 years, data were collected again through the same procedure. The directly affected participants were not asked to participate in the 4-year survey, as a follow-up of these children and their families was undertaken by the hospital that had organised the acute crisis intervention [20]. The present study was commenced in 2008, 20 years after the event, when addresses for 102 former pupils (57 men and 45 women), now aged 33 years, could be retrieved, and to whom a survey was sent by mail.

### Participants

The participants were defined as either directly or indirectly affected by the incident. The directly affected participants were those who were involved in the bus crash on the school outing, and the indirectly affected participants were all children of the same age in the affected school who did not participate in the school outing. Previous studies [17,18,22] determine no difference in acute or chronic posttraumatic stress between near-miss subjects (who were supposed to be at the site of the disaster but for some reason were not there) and those who were not supposed to be at the site and were not there; thus, the children in the affected class who did not participate in the school outing were included in the indirectly affected group.

**Figure 1:**
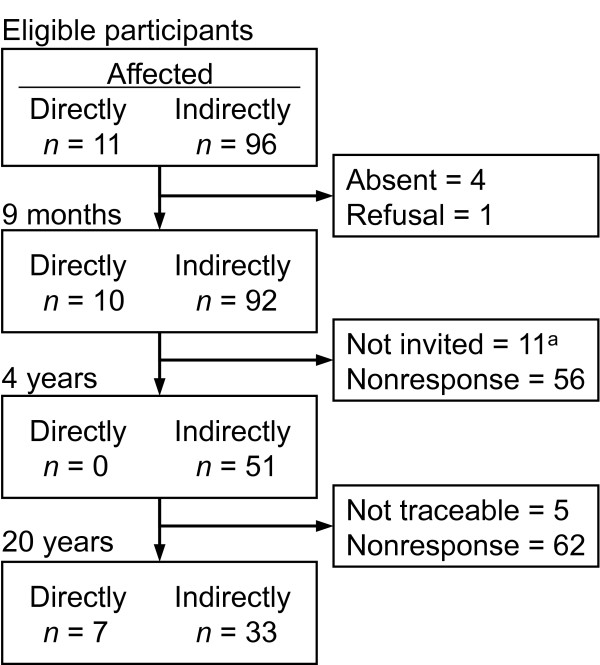
**The total number of eligible participants and the number of respondents in each survey**. ^a^All directly affected.

In the survey 9 months after the accident, 102 (95%) children responded: 55 boys and 47 girls (Figure 1). After 4 years, 51 (48%) children responded, 24 boys and 27 girls: none of the directly affected children were included in follow-up data collection. After 20 years, 40 (39%) of the now 33 year-old participants responded, 19 men and 21 women. There were 33% men and 48% women who responded, the difference was not statistically significant (χ^2 ^= 2.51, *p *= .16). The majority had a degree from high school (*n *= 19) or university (*n *= 18) and were currently employed (*n *= 37). There were 35 who were in a relationship, and 15 had children. Educational, marital, and employment status were similar for the directly and indirectly affected.

### Measures

The questionnaires distributed at 9 months and 4 years were compiled based on a study of a school bus accident in Israel in 1985 [17]. The questionnaire comprised (a) nineteen dichotomous (yes/no) items covering psychological reactions during the preceding three weeks (e.g., *I have had nightmares about the bus crash*); (b) 4 items about social and professional support received; and (c) 16 items probing the participants' interest in and preferences for future help. The 19 items on posttraumatic stress were derived by Milgram et al. [17] from clinical literature on posttraumatic stress and bereavement reactions in children, and eight of the items were identical to the Child PTSD Reaction Index developed by Pynoos et al. [22]. In this study, these eight items were analysed as single items: nightmares, avoidance, fear, worry or anxiety, intrusions, concentration difficulties, sadness, and loss of interest in daily activities. In addition, one item assessing guilt was analysed (*I have felt guilty about the injury or death of others*).

The Impact of Event Scale-Revised (IES-R) [23,24] was used to assess posttraumatic stress after 20 years. The IES-R comprises 22 items assessing the frequency of intrusion, avoidance, and hyperarousal reactions during the previous week, with regard to a specific event. As in the original IES [25], the items were coded 0, 1, 3, and 5, where 0 equals *no symptoms *and 5 equals *high frequency of symptoms*. All items were summed to create a total symptom score (total score 0-75): Cronbach's alpha for the IES-R was 0.96.

The 12-item General Health Questionnaire (GHQ-12) [26,27] assesses general mental health and focuses on inability to undertake normal functions and the appearance of new and distressing phenomena. GHQ-12 is sensitive to short-term disorders, but not enduring attributes of the respondent [28], and is reliable and valid as a screening tool in community samples in different cultural contexts [29,30]. A sum score of the Likert-coded items (0-1-2-3) was used for the calculation of median values: Cronbach's alpha was 0.80 for the GHQ-12.

The Complicated Grief Index (CGI) [31] comprises nine items from the Inventory of Complicated Grief (ICG) [32] and constitutes the concept of complicated, unresolved grief [33,34]: a yearning for and preoccupation with the deceased that interrupts normal activities; trouble accepting the loss; detachment; bitterness; loneliness; feeling part of one's self died and that life is empty; and, loss of security or safety. The respondent indicates the frequency of symptoms during the previous month on a 5-point scale ranging from 0 (*almost never*) to 4 (*always*). A complicated grief reaction is indicated if the respondent replies with *often *or *always *to at least 5 symptoms (i.e., a score of ≥ 15 points), one of which has to be yearning: Cronbach's alpha was 0.92 for the CGI.

Negative life events were assessed after 20 years by an inventory of 13 items [35]. The participants were asked if and when they had experienced any negative life events (i.e., disaster, war/terror, death of a family member or close friend, threat to physical/psychological integrity, serious disease or injury to self or family member, accident, divorce, serious financial problems). Participants were asked to rate the impact of the event on a four-point scale (*none, small, moderate*, and *great*). A total score was achieved by summing the number of events with a moderate or great impact. Cronbach's alpha was not computed, as it is not meaningful for these types of inventories.

In addition, after 20 years, information on whether the participants had received psychological or psychopharmacological treatment was collected, and the survey contained open-ended questions to collect the participant's views on e.g., whether the event still affected their daily lives.

### Statistical Analysis

Only data on group level had been retained from the first two surveys, i.e., only the number of participants in the directly and indirectly affected groups that had endorsed the items on psychological reactions. A risk ratio (RR) with 95% confidence interval and Fisher's Exact Test was used to assess the size and significance of the differences in single-item symptoms between the directly and indirectly affected groups after 9 months. As 9-month and 4-year data for each individual was unavailable, Wild and Seber's test [36] for paired proportions was used to assess changes in prevalence of psychological reactions over time. Spearman's rho was used for correlations between continuous variables. Mann-Whitney's *U*-test was used to test the significance of differences in IES-R, GHQ-12, and CGI between the directly and indirectly affected groups after 20 years. The assumptions for the tests in the analyses were fulfilled, although the distributions of IES-R and CGI scores were positively skewed and the GHQ-12 scores were symmetric around the mean, which precluded computing confidence intervals for the skewed variables. Median (*Mdn*) and interquartile range (*IQR*) are reported, and for comparative purposes mean and standard deviation for IES-R are also reported. The level of significance was set to alpha = 0.05, two-tailed. The participants' answers to open-ended questions in the 20-year survey were transcribed verbatim and the content was coded into categories. Data analysis was performed with SPSS version 16.0.1 for Windows (SPSS, 2007).

### Ethical Approval

The initial study was approved by the Ethical Committee at the Karolinska Institutet, Stockholm, Sweden, and the present follow-up study was approved by the Regional Ethical Review Board in Uppsala, Sweden, record no. 2008/358.

## Results

### After 9 Months

The reactions endorsed by at least 10% of the participants at 9 months are presented in Table 1. Less than 10% of the sample experienced concentration difficulties, worry or anxiety, and loss of interest in daily activities. A majority of the directly affected participants experienced sadness, guilt, nightmares, and tried to avoid thinking of the accident. The indirectly affected participants differed from the directly affected in that they did not experience guilt or nightmares to any great extent (Table 1).

**Table 1:** Psychological reactions at three assessments after a school-bus crash

	9 Months				4 years	20 years
			
Reactions	Total (*n *= 102)	Directly affected (*n *= 10)	Indirectly affected (*n *= 92)	RR [95%CI]	Indirectly affected (*n *= 51)	Indirectly affected (*n *= 33)
Sadness	70 (69%)	9 (90%)	61 (66%)	1.2 [0.7-2.0]	21 (42%)*	6 (18%)*
Avoidance	60 (59%)	8 (80%)	52 (59%)	1.4 [0.99-2.0]	15 (29%)**	4 (12%)
Fear^a^	23 (23%)	2 (20%)	21 (23%)	0.99 [0.7-1.4]	11 (21%)	-
Guilt	19 (18%)	6 (60%)	12 (13%)	4.6 [2.2-9.6]**	13 (25%)	3 (9%)*
Intrusions	17 (17%)	1 (10%)	16 (17%)	0.93 [0.7-1.2]	9 (17%)	5 (15%)
Nightmares	13 (13%)	5 (50%)	8 (9%)	5.8 [2.3-14.2]**	6 (12%)	1 (3%)*

*Note*. The risk ratio (RR) with 95% confidence interval (CI) compared the prevalence of reactions at 9 months in directly and indirectly affected groups. Asterisks at 4 and 20 years indicate a statistically significant difference in prevalence of reactions in the indirectly affected participants compared with the previous assessment.

^a^Not assessed at 20 years.

*p< .05. **p < .01.

There were 67 (66%) children who had sought support from friends, parents or teachers: 36 (77%) of the girls and 31 (56%) of the boys. One-third of the girls (*n *= 16) and one-quarter of the boys (*n *= 13) felt they had not recovered fully from the event. However, there were 8 (8%) who endorsed they would like to meet a professional to talk about their feelings about the accident: 7 (15%) of the girls and 1 (2%) of the boys.

### After 4 Years

The directly affected group did not participate in this wave of data collection. There were 24 (47%) of the 51, now 15-16 years old, indirectly affected participants who had experienced no upsetting thoughts during the past year about the accident: 17 (63%) of the boys and 7 (29%) of the girls (*RR *= 2.2 [1.1-4.3], *p *= .025). Seven (29%) girls, but no boys, reported bus travel anxiety. Furthermore, 12 (50%) of the indirectly affected participants endorsed they would like to meet a professional to talk about the bus crash.

Wild and Seber's [36] paired proportions test, used to assess the changes in psychological stress reactions in the indirectly affected group between 9 months and 4 years, indicated the prevalence of sadness decreased (*z *= 1.79, *p *= .037), and had further decreased after 20 years. The prevalence of avoidance decreased between 9 months and 4 years (*z *= 2.28, *p *= .011). The proportion of indirectly affected experiencing feelings of guilt did not decrease between 9 months and 4 years (*z *= 1.39, *p *= .082). Nightmares did not decrease between 9 months and 4 years (*z *= 0.47, *p *= .32). No difference was found between 9 months and 4 years in feelings of fear when thinking about the accident (*z *= 0.22, *p *= .41).

### After 20 Years

The directly affected group reported some posttraumatic stress reactions after 20 years (Table 2), with fewer reactions in those indirectly affected (Mann-Whitney *U *= 195, *p *= .003). No difference was identified between directly and indirectly affected participants regarding general mental health assessed by the sum score of GHQ-12 (*U *= 73, *p *= .14). For those who had been bereaved of close friends, parents or classmates (*n *= 33), there was a strong positive correlation between posttraumatic stress and complicated grief (*rho*_31 _= .78, *p *< .001). There was no difference for complicated grief between the directly and indirectly affected (*U *= 74.5, *p *= .45).

**Table 2:** Posttraumatic stress, complicated grief, and general mental health 20 years after a school-bus crash

		Impact of Event Scale-Revised					CGI	GHQ-12
				
		Total		Intrusion	Avoidance	Hyperarousal		
				
Participants	*N*	*M (SD)*	*Mdn (IQR)*	*Mdn (IQR)*	*Mdn (IQR)*	*Mdn (IQR)*	*Mdn (IQR)*	*Mdn (IQR)*
Directly affected	7	21 (17)	17 (29)	7 (12)	7 (11)	3 (9)	3 (8)	12 (7)
Indirectly affected	33	8 (15)	3 (8)	3 (6)	0 (3)	0 (0)	1 (4)	9 (6)
Total	40	10 (16)	4 (11)	3 (8)	0 (4)	0 (1)	1 (6)	9 (5)

*Note*. CGI = Complicated Grief Index. GHQ-12 = General Health Questionnaire-12.

Wild and Seber's [36] paired proportions test indicated the prevalence of sadness in the indirectly affected group decreased between 4 and 20 years (*z *= 1.78, *p *= .038). The decrease in prevalence of avoidance approached significance (*z *= 1.53, *p *= .064), which was also true for prevalence of feelings of guilt (*z *= 1.58, *p *= .057). Nightmares did not decrease between 4 and 20 years (*z *= 1.33, *p *= .091). For the directly affected group, no tests were carried out due to the low power. However, between 9 months and 20 years, the proportion of directly affected experiencing sadness changed from 90% (9 months) to 14% (20 years): for the same follow-up times, avoidance decreased from 75% to 29%, intrusions decreased from 14% to 29%, nightmares decreased from 50% to 0%, and feelings of guilt decreased from 63% to 14%.

During the 20 years, other negative life events or receiving treatment may have influenced the participants' posttraumatic stress or general mental health. The participants had experienced a median of one negative life event with a moderate or great impact (*IQR *= 2, range 0-6). The median and IQR were the same for both the directly and indirectly affected groups. The directly affected had experienced 0-2 negative life events, whereas the indirectly affected had experienced 0-6 events. The events that more than 10% of the sample had experienced were: serious disease or injury to a family member (*n *= 14), bereavement of a close relative or friend (*n *= 11), bereavement of a parent (*n *= 7), serious physical/psychological violence (*n *= 6), and traffic/other accident (*n *= 5). The association between number of negative life events and posttraumatic stress was small and not significant (*rho*_38 _= .29, *p *= .066), whereas, the association with general mental health was moderate (*rho*_38 _= .40, *p *= .011).

Whether having received treatment was related to current posttraumatic stress or general mental health was explored. Ten participants, whereof three were directly and seven were indirectly affected, had received psychological or psychopharmacological treatment during the past 10 years for affective, anxiety, or eating disorders. Participants who had received treatment (*n *= 10) had IES-R *Mdn *(*IQR*) = 12 (25) and GHQ-12 *Mdn *(*IQR*) = 7 (4), whereas, those who had not received treatment (*n *= 30) had IES-R *Mdn *(*IQR*) = 3 (7) and GHQ-12 *Mdn *(*IQR*) = 13 (6). The differences in medians were statistically significant for both posttraumatic stress (*U *= 78, *p *= .02) and for general mental health (*U *= 53, *p *= .002).

Twenty years later, 11 out of 40 participants reported they were still influenced by the accident, whereof seven participants were directly affected. The eleven participants were asked to describe how they were influenced by the event, and to regard both negative and positive consequences. No apparent differences in meaning were discerned between directly and indirectly affected participants. The examples presented herein serve as illustrations of how the event still affected the participants. Four of the eleven participants described an increased influence of the event when they became parents: one participant expressed that he/she "every day consider that my children might die of something, suffocate, being run over by a motor vehicle, murdered." Three participants stated they had learned to cherish valuable things in life: one participant had "realised the importance of caring for relations with others." Two expressed survivor guilt: one stated that he/she "think[s] about [the crash] every time I hear about other accidents. I wonder if I was worth surviving."

## Discussion

The limitations inherent in the small sample and the attrition precluded precise inferences. Nevertheless, given the dearth of published long-term studies on psychological consequences for children involved in major traffic accidents, the findings could contribute to further research. The initial load on psychological resources was apparently high. After four years, some psychological reactions had decreased in the indirectly affected group, and further continued up to 20 years after the incident. At this point, the now adult participants who were directly affected reported mild posttraumatic stress, and indirectly affected participants endorsed almost no posttraumatic stress reactions. No difference in general mental health or in complicated grief was observed between the groups. Complicated grief and posttraumatic stress reactions were correlated after 20 years, and mostly participants who were directly affected experienced the event still affected their daily lives.

In the first survey after 9 months, there was a substantial proportion who reported sadness and avoidance reactions in both the directly and indirectly affected groups, and the directly affected had more feelings of guilt and nightmares. Intrusion is considered a hallmark symptom of PTSD, and although no assessment was made as to whether the children suffered from PTSD, the 9% prevalence of nightmares in the indirectly affected group paralleled the 9-month 6% prevalence of PTSD found in indirectly affected schoolchildren after a bus-train collision in Israel [17]. In the present study, 8% of the children stated they would like to meet a professional to talk about their feelings about the accident.

After 4 years, about half of the indirectly affected participants reported no upsetting thoughts during about the bus crash the past year; however, the remainder felt that they would like to meet a professional to talk about the event. In a 7-year follow-up after an Israeli bus and train collision [18], 12-17% of the then 20-years old conscripts who had been both directly and indirectly exposed had sought military mental health services, compared with 1% of children from a nearby school. These findings substantiate help-seeking behaviour is also expected to increase in indirectly affected for several years after a large RTA. In this study, only changes in sadness and avoidance were determined between 9 months and 4 years, whereas and there were only small or no changes for intrusions and nightmares, and feeling guilt or fear when thinking of the bus crash. Therefore, the findings herein were similar to clinical observations after a mudslide engulfed a primary school [37]: during the first four years, the most common symptoms reported by the children to their general physician were sleeping difficulties, nervousness, avoidance, instability and enuresis.

After 20 years, the directly affected group reported some chronic posttraumatic stress reactions, and these were still markedly higher than the indirectly affected who reported minimal chronic reactions. In the indirectly affected group, intrusions appeared more long-standing than avoidance and hyperarousal reactions. In a 33-year follow-up of children affected by a mudslide [16], significant posttraumatic stress remained in a quarter of the sample, whereas, the rates of other psychopathological disorders were not elevated. However, it can only be speculated as to whether the higher symptom load after the mudslide disaster, compared with the results presented here, is due to a ubiquitous threat of another mudslide imposed on those children, or the result of successful acute support interventions after the bus crash [20]. Posttraumatic stress reactions, but not general psychopathology, can be prolonged into adulthood in children and adolescents who have been exposed to single traumatic events [16,18,38], which was supported by the results of this study. Negative life events, other than the bus crash, were positively associated with decreased general mental health but not with posttraumatic stress after 20 years.

Measures of posttraumatic stress in bereaved trauma samples victims may be confounded by prolonged grief reactions [39], and traumatic bereavement is associated with worse long-term outcome in both children [40] and adults [35]. In this study, grief and posttraumatic stress were closely intertwined, and further studies could disentangle the long-term consequences of traumatic stress and traumatic bereavement.

This study suffered from several limitations. First, the sample size was small, especially for the directly affected group, as only 11 children survived the bus crash. The inherent difficulties would have been reduced if the response rate for the indirectly affected group had been higher: only 33 responded out of 91 individuals traced from this group. This introduced possible response bias, i.e., the respondents were a biased sample of all indirectly affected eligible participants. It has been suggested those least affected are less inclined to respond [41]. Thus, a response bias would have attenuated the differences between the indirectly and directly affected groups. Second, as only aggregate data were retained from the first two surveys, a comparison of respondents and non-respondents in the indirectly affected group based on previous assessment of PTSS was precluded. Further, in consideration of the age of the participants and the setting in which the study was conducted only single items were chosen in the first two surveys for identifying affective and anxiety reactions, These items were derived from a reliable and valid questionnaire [17,22], however, as the items have not been empirically tested, the validity and reliability is uncertain, although face validity may not have been compromised.

Although the assessment of treatment effects was beyond the scope of this study, the results suggested participants who had received treatment since the event had more posttraumatic stress reactions and worse general mental health than those who had not received treatment. This corresponded to previous findings [35,42], and might be due to direct involvement predisposing individuals towards seeking professional help [18], or that those who seek treatment have a higher acute symptom load. As such, the results implied that receiving treatment was not as a result of improved subsequent mental health, but as an effect of worse prior mental health. That treated subjects report worse mental health needs addressing in further studies.

## Conclusions

Although the limitations prevent assertive conclusions from this study, the findings supports previous research on long-term psychological consequences for children involved in major road traffic accidents in that while general mental health problems are not elevated many years later, posttraumatic stress reactions can persist long into adulthood.

## Competing interests

The authors declare that they have no competing interests.

## Authors' contributions

FKA participated in the design of the study and acquisition of data, performed the data analysis and drafted the manuscript. PAR and TL created, and participated, in the design of the study, collected data and helped to draft the manuscript. All authors read and approved the final manuscript.
